# A two-phase randomized study of oliceridine versus sufentanil for gastrointestinal endoscopy

**DOI:** 10.3389/fphar.2026.1853405

**Published:** 2026-05-28

**Authors:** Dengyun Hu, Panpan Ding, Junjie Liu, Kaixiang Cao, Hongyun Bi, Jinfeng Bao, Linghui Huang, Tingting Zhang, Xin Wang

**Affiliations:** 1 Graduate School of Bengbu Medical University, Bengbu, Anhui, China; 2 Department of Anesthesiology, The Second People’s Hospital of Hefei, Hefei Hospital Affiliated to Anhui Medical University, Hefei, Anhui, China

**Keywords:** ED_95_, gastrointestinal endoscopy, oliceridine, painless, sufentanil

## Abstract

**Background:**

Oliceridine is a novel G protein-biased μ-opioid receptor (MOR) agonist. It provides analgesic effects and reduces opioid-related adverse events (ORAEs). It has shown good efficacy in acute pain management. However, compared with traditional opioids, its optimal dosage and safety in gastrointestinal endoscopy have not been clearly established. We aimed to determine the 95% effective dose (ED_95_) of oliceridine and sufentanil in gastrointestinal endoscopy and simultaneously evaluate their therapeutic effects.

**Methods:**

This two-phase study was designed to evaluate the clinical effective dose and clinical safety of oliceridine. In the first phase, we used the modified Dixon’s up-and-down method to determine the ED_50_ and ED_95_ of oliceridine and sufentanil during sedated gastrointestinal endoscopy. In Phase 2, 160 patients undergoing gastrointestinal endoscopy were randomized to receive oliceridine or sufentanil at the predefined doses in combination with propofol titrated to a standardized depth of sedation. The primary outcomes were time to discharge and incidence of respiratory depression. Secondary outcomes included recovery profiles and adverse events.

**Results:**

In the first phase, we determined the ED_95_ to be 14.52 μg/kg (95% CI: 12.44–24.23) for oliceridine and 0.048 μg/kg (95% CI: 0.044–0.071) for sufentanil. In the second phase, the oliceridine group achieved a significantly shorter discharge time (13.0 min vs. 15.0 min, *P* = 0.044) and reached the recovery criterion (modified Aldrete score ≥9) more quickly (5.0 min vs. 6.0 min, *P* = 0.013). Importantly, the incidence of respiratory depression was markedly lower in the oliceridine group (7.5% vs. 20%, *P* = 0.039). Other adverse events were similar between groups.

**Conclusion:**

At doses derived from a sequential dose-finding design, oliceridine combined with propofol was associated with a lower observed incidence of respiratory depression and modestly faster recovery compared with sufentanil during gastrointestinal endoscopy in low-risk patients. Given the methodological limitations of dose estimation and the limited power for safety outcomes, these findings should be interpreted cautiously and warrant confirmation in larger studies.

**Clinical Trial Registration:**

Identifier ChiCTR2500095827.

## 1 Introduction

Changes in modern lifestyles and the aging of the global population are driving a continuous increase in the incidence of gastrointestinal (GI) diseases, posing a significant threat to public health and imposing a heavy health-economic burden. According to the latest statistics from the World Health Organization (WHO) and the International Agency for Research on Cancer (IARC), there are approximately 20 million new cancer cases and 9.7 million deaths worldwide annually (Bray et al., 2024). The Global Burden of Disease (GBD) 2021 study further reveals that GI cancers account for 5.26 million new cases and 3.7 million deaths, representing nearly one-third of all global cancer fatalities, with colorectal and gastric cancers being the primary contributors (Danpanichkul et al., 2024). These findings highlight the prominent position of GI cancers in the global cancer burden. Consequently, strengthening the control of risk factors, promoting early screening, and optimizing treatment strategies are crucial for mitigating the future burden of these diseases. In clinical practice, gastrointestinal endoscopy remains the gold standard for diagnosing these conditions. It provides direct mucosal visualization and allows targeted biopsy with histopathological evaluation, which significantly enhances diagnostic accuracy (Winawer et al., 1993; King et al., 2025; W et al., 2024).

During upper gastrointestinal endoscopy, the passage of the endoscope through the oropharynx triggers intense physical stimulation, frequently leading to pain, nausea, vomiting, and even a sensation of asphyxia, which severely impairs patient compliance (Zhou and Li, 2023; Abraham et al., 2002). Furthermore, patient discomfort during unsedated gastroscopy significantly limits the thoroughness of endoscopic observation, potentially leading to the missed diagnosis of subtle lesions such as early esophageal cancer and increasing the risk of procedural accidents and unintended injuries (Hao et al., 2020). Therefore, procedural sedation can enhance patient tolerance, ensure diagnostic precision, and minimize accidental risks (Early et al., 2018).

Colonoscopy entails significant mechanical stressors, including colonic distension from insufflation, mesenteric traction during scope navigation, and direct mucosal irritation. As highlighted in a recent review (Chudziński et al., 2025), pain perception during endoscopy is a multidimensional process modulated by physiological, anatomical, and psychological factors, involving both peripheral nociceptor activation and central sensitization. Evidence suggests that discomfort resulting from inadequate sedation not only severely limits the comprehensiveness of observation and increases the risk of missing neoplastic lesions but may also necessitate premature termination of the procedure. Furthermore, negative procedural experiences can significantly deter patients from future surveillance, directly undermining the long-term effectiveness of colorectal cancer prevention programs. Thus, implementing an appropriate sedation regimen is critical for optimizing the quality of colonoscopy.

With the widespread application of painless gastrointestinal endoscopy, sedation strategies have exhibited a significant evolutionary trend. Although current clinical guidelines still exhibit significant divergence and lack unified, high-quality evidence regarding the choice of specific sedatives, frontline clinical practice is evolving toward a multi-drug ‘balanced sedation’ paradigm to address the respiratory and hemodynamic challenges posed by propofol monotherapy (Dossa et al., 2021). The latest systematic review and network meta-analysis demonstrated that propofol–opioid combinations remain the commonly referenced regimen and achieved the highest sedation success rate in gastrointestinal endoscopy (Li et al., 2025). This regimen relies on traditional μ-opioid receptor agonists and provides adequate sedation and analgesia. Although clinically effective, such combinations are often associated with adverse effects. Respiratory depression and postoperative nausea and vomiting (PONV) are common. These adverse effects can impact patient comfort, safety, and recovery quality (Li et al., 2025; Mercadante, 2019). Therefore, optimizing treatment regimens to balance efficacy and safety is imperative.

Oliceridine is a novel selective μ-opioid receptor (MOR) agonist. It works through a G protein-biased mechanism, preferentially activating the G protein pathway and minimizing β-arrestin recruitment. This unique action provides analgesia comparable to traditional opioids and reduces the incidence of adverse events such as respiratory depression and PONV (Daksla et al., 2023; Soergel et al., 2014a). Pharmacokinetically, according to FDA evaluation documents, oliceridine demonstrates a rapid onset of 2 to 5 min and a predictable plasma clearance profile. Based on these established PK characteristics, we postulate that its pharmacodynamic window theoretically aligns well with the requirements of short gastrointestinal endoscopic procedures, which typically last only 15 to 30 min (Food and Drug Administration, 2026; Food and Drug Administration (FDA), 2018). Although oliceridine has been approved for acute pain management, its use in procedural sedation, particularly its interaction mechanism with propofol, is still being investigated. Currently, clinical evidence defining the optimal analgesic dose of oliceridine for gastrointestinal endoscopy is still limited. To address this knowledge gap, we conducted this two-phase investigation. First, we employed a sequential up-and-down method to determine the 95% effective dose (ED_95_) of oliceridine. Second, we compared its clinical profile with sufentanil under standardized diagnostic endoscopic conditions. We anticipate that the results will help clarify the clinical value and therapeutic potential of oliceridine for procedural analgesia.

The inclusion criteria were as follows: 1) age 18–65 years; 2) American Society of Anesthesiologists (ASA) physical status I–II. ASA II patients were limited to those with stable, well-controlled comorbidities (e.g., hypertension or diabetes); 3) body mass index (BMI) of 18.5–27.9 kg/m^2^; 4) scheduled for elective painless gastrointestinal endoscopy, excluding therapeutic procedures; 5) ability to understand and voluntarily sign the informed consent form, and willingness to comply with the study protocol.

The exclusion criteria were as follows: 1) unwillingness or inability to complete the entire study; 2) allergy or contraindication to opioids, propofol, or any study drug components; 3) severe cardiac, hepatic, or renal dysfunction; 4) history of alcohol abuse or psychiatric disorders, pregnancy, or lactation; 5) chronic pain conditions requiring long-term analgesic therapy. 6) a history of opioid abuse; and 7) inflammatory bowel disease (e.g., active Crohn’s disease).

## 2 Materials and methods

### 2.1 Study design and ethics

**FIGURE 1:**
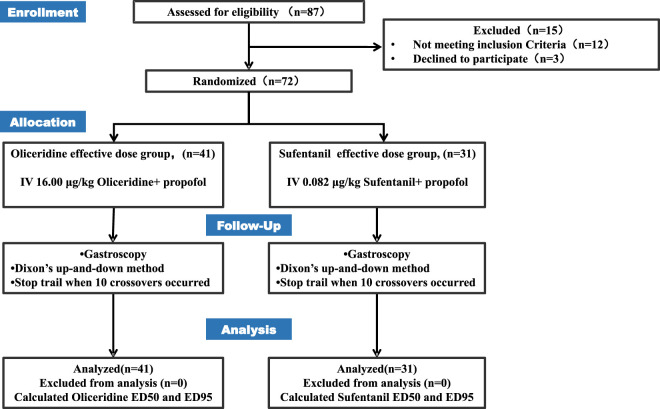
Patient enrollment and allocation for Phase one (Dose-finding). Of the 87 patients assessed, 72 were randomized to receive either oliceridine (n = 41) or sufentanil (n = 31) combined with propofol. Dosages were adjusted using Dixon’s up-and-down method until 10 crossovers occurred to calculate the ED_50_ and ED_95_. All 72 randomized patients were included in the final analysis.

**FIGURE 2:**
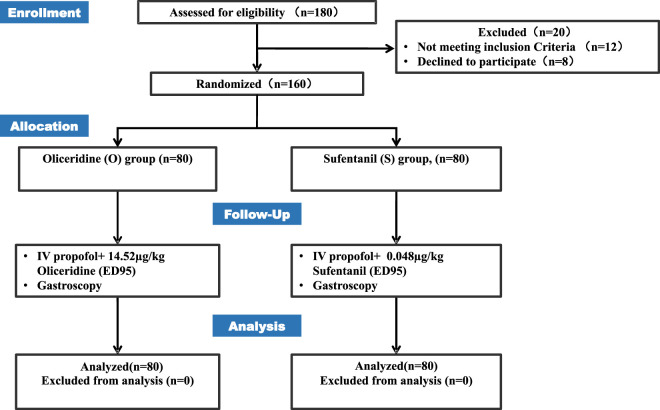
Patient enrollment and allocation for Phase two (RCT). Of the 180 patients assessed, 160 were randomized equally (n = 80 per group) to receive either oliceridine (14.52 μg/kg) or sufentanil (0.048 μg/kg) at their respective ED_95_ doses combined with propofol. All 160 patients completed the gastroscopy and were included in the final analysis.

This single-center, prospective study consisted of two sequential phases: a dose-finding phase (Phase 1, Figure 1) and a double-blind, randomized controlled trial (Phase 2, Figure 2). The protocol was approved by the Ethics Committee of Hefei Second People’s Hospital (Approval No. 2024-Research-186). The study was registered at the Chinese Clinical Trial Registry (ChiCTR2500095827). Written informed consent was obtained from all participants. Note: The registry primarily details the confirmatory Phase two RCT; Phase one was conducted as an adaptive internal pilot study under the same ethical approval to calibrate the dosing parameters for the main trial.

### 2.2 Anesthesia protocol (standardized for both phases)

All patients fasted for at least 8 h and from clear liquids for 4 h prior to the procedure. All patients received 10 mL of dyclonine hydrochloride mucilage as a topical local anesthetic before entering the procedure room. No other pharmacological premedications were administered prior to the induction of anesthesia. Upon establishing intravenous access, a maintenance infusion of normal saline was initiated at a rate of 40–60 drops/min. Patients were then positioned in the left lateral decubitus position and received supplemental oxygen (3 L/min) via nasal cannula. Routine physiological monitoring included electrocardiography (ECG), non-invasive blood pressure (NIBP), and pulse oximetry (SpO_2_). The patient received an intravenous bolus of assigned opioid. Each bolus was administered over more than 10 s. After a 2-min interval to allow for drug onset, propofol was titrated in 20–30 mg increments every 10 s until loss of consciousness. The strict induction endpoint was defined by the loss of eyelash reflex, a Modified Observer’s Assessment of Alertness/Sedation (MOAA/S) score ≤ 1, and a bispectral index (BIS) value between 50 and 60. This rigorous standardization was vital to ensure that outcomes were not confounded by discrepancies in sedation depth. Maintenance was achieved via continuous propofol infusion (50–150 μg/kg/min) adjusted to maintain a BIS of 50–60. The mean arterial pressure (MAP), heart rate (HR), pulse oximetry (SpO_2_), and respiratory rate (RR) were assessed and documented.

### 2.3 Phase 1: determination of ED_95_

A modified Dixon’s up-and-down sequential allocation method was employed for dose determination. Based on the results of a pre-experimental pilot study, the initial doses were established as 16.00 μg/kg for oliceridine and 0.082 μg/kg for sufentanil. Dosing for each subsequent patient was adjusted by a fixed ratio of 1.2 based on the “response” of the preceding participant, defined as coughing, gross purposeful movement, or a swallowing reflex during endoscope insertion. To enhance the precision of the ED_95_ and population variance estimates, testing continued until 10 success-failure crossover points were achieved in each group, rather than the traditional 6 crossovers often used for ED_50_ estimation (Pace and Stylianou, 2007). The ED_50_ and ED_95_ were calculated using probit regression analysis.

### 2.4 Phase 2: randomized controlled trial

#### 2.4.1 Randomization and blinding

160 patients were randomized (1:1) using a computer-generated sequence into Group O (Oliceridine) or Group S (Sufentanil). An independent nurse, not involved in patient care, prepared the study drugs in identical 10 mL syringes labeled only with the study ID to ensure double-blinding of the anesthesiologist and endoscopist.

Intervention: Group O: Received Oliceridine at the calculated ED_95_; Group S: Received Sufentanil at the calculated ED_95_.

#### 2.4.2 Outcomes

The primary efficiency endpoint was the time to discharge, defined as the interval from the completion of the procedure to discharge from the Post-Anesthesia Care Unit (PACU). The primary safety endpoint was the incidence of respiratory depression, defined as a RR < 8 breaths/min (measured via thoracic impedance and continuous clinical observation) or SpO_2_ level < 90%, with either condition lasting for more than 10 s. Sample size was calculated based on preliminary pilot data for the primary efficiency endpoint (time to discharge: 14.17 ± 4.13 min vs. 16.42 ± 4.34 min). To achieve 90% power (α = 0.05) to detect these clinical efficiency differences, 75 patients per group were required. By prioritizing the efficiency metric for power analysis, we ensured a sufficient sample size to capture subtle differences in clinical workflow while simultaneously monitoring for safety signals. Secondary measures included the incidence of [defined as hypotension (MAP < 60 mmHg) or bradycardia (HR < 50 beats/min)], total propofol consumption, and the incidence of venous injection pain. Additionally, we assessed the severity of postoperative abdominal pain [measured by a 0–100 mm visual analogue scale (VAS) at PACU discharge], the time to reach a modified Aldrete score ≥ 9 (indicating early neurological recovery), and the incidence of postoperative nausea and vomiting (PONV). Hemodynamic parameters (MAP, HR, RR, and SpO_2_) were continuously monitored and recorded at five predefined time points: T0 (baseline, supine position), T1 (2 min after opioid administration), T2 (endoscope entry into the main anatomical lumen), T3 (endoscope withdrawal from the main anatomical lumen), and T4 (full recovery of consciousness).

### 2.5 Statistical analysis

All statistical analyses and data visualization were performed using SPSS version 26.0 (IBM Corp., Armonk, NY, United States) and GraphPad Prism version 9.0 (GraphPad Software, San Diego, CA, United States).

Sample size was calculated based on preliminary pilot data for time to discharge, which was 14.17 ± 4.13 min in the oliceridine group and 16.42 ± 4.34 min in the sufentanil group. To achieve a statistical power of 90% (α = 0.05, two-tailed), 75 patients per group were required. Accounting for potential dropouts, 80 patients were enrolled per group. Additionally, a post-hoc power analysis was conducted using G*Power software (version 3.1.9.2; Heinrich-Heine-Universität Düsseldorf, Germany) to evaluate the statistical power for detecting differences in the primary safety endpoint (respiratory depression).

The Shapiro-Wilk test was utilized to assess the normality of continuous variables. Normally distributed continuous data were expressed as mean ± standard deviation (SD) and compared using the independent-samples Student’s t-test. Non-normally distributed data were presented as median and interquartile range (IQR) and analyzed using the Mann-Whitney U test. Categorical data were presented as frequencies and percentages (n, %) and compared using Pearson’s Chi-square test or Fisher’s exact test, as appropriate.

For the repeated hemodynamic and respiratory measurements across predefined time points (T0–T4), group comparisons were performed using the two-sample t-test or Mann-Whitney U test. To mitigate the risk of Type I errors from multiple comparisons, a Bonferroni correction was applied, setting the threshold for statistical significance at *P* < 0.01 for these specific metrics. In Phase 1, ED_50_ and ED_95_, along with their 95% confidence intervals (CIs), were calculated using Probit regression analysis. For all other analyses, a two-sided *P*-value < 0.05 was considered statistically significant.

## 3 Results

### 3.1 Phase 1: effective dose determination

**FIGURE 3:**
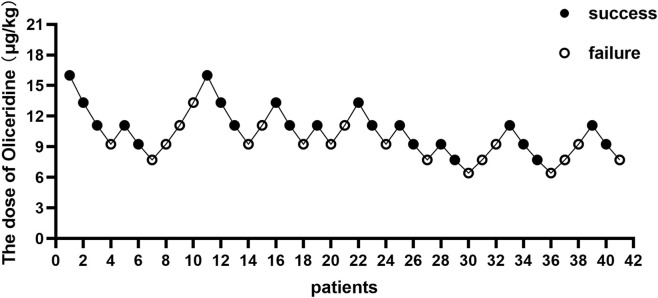
Experimental trajectories for determining the ED_50_ and ED_95_ of oliceridine via Dixon’s up-and-down method. Solid circles represent success (effective motor suppression), and open circles represent failure.

**FIGURE 4:**
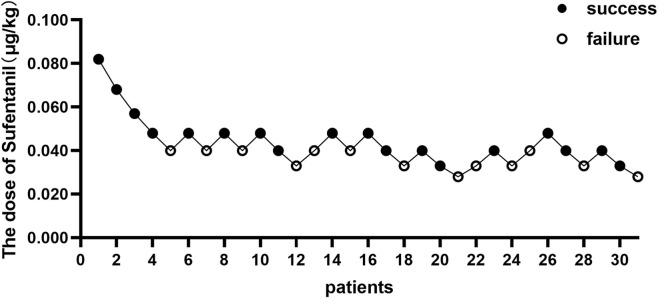
Experimental trajectories for determining the ED_50_ and ED_95_ of sufentanil via Dixon’s up-and-down method. Solid circles represent success (effective motor suppression), and open circles represent failure.

In Phase one of the study, a total of 72 patients were enrolled to evaluate the dose-response relationship, with 41 patients allocated to the oliceridine group and 31 patients to the sufentanil group. The experimental trajectories of individual patient responses to motor suppression, assessed using Dixon’s up-and-down sequential allocation method, are illustrated in Figures 3, 4.

Statistical analysis revealed that for oliceridine, ED_50_ was 9.80 μg/kg, and ED_95_ was calculated at 14.52 μg/kg (95% CI: 12.44–24.23 μg/kg). For sufentanil, the ED_50_ was 0.038 μg/kg, and the ED_95_ was determined to be 0.048 μg/kg (95% CI: 0.044–0.071 μg/kg). Based on these findings, the determined ED_95_ values (14.52 μg/kg for oliceridine and 0.048 μg/kg for sufentanil) were adopted as the fixed administration doses for the subsequent efficacy and safety evaluations in Phase 2.

### 3.2 Phase 2: clinical efficacy and safety

#### 3.2.1 Patient characteristics and anesthesia details

A total of 160 patients were ultimately included in the final analysis, with 80 patients allocated to Group O (oliceridine) and 80 patients to Group S (sufentanil). While gastroscopy and colonoscopy have different clinical profiles, they were included to reflect the real-world ‘balanced sedation’ paradigm in our outpatient endoscopy unit. Notably, the distribution of examination types was statistically comparable between Group O and Group S (32/7/41 vs. 34/5/41, *P* = 0.821), ensuring that the procedure type did not confound the primary efficacy and safety evaluations. There were no statistically significant differences between the two groups in terms of baseline demographics and procedure characteristics (Table 1). Furthermore, the total consumption of propofol was comparable between Group O (197.5 ± 52.6 mg) and Group S (192.9 ± 52.1 mg) (*P* = 0.581, Table 3), indicating a consistent depth of sedation across both groups throughout the procedure.

**TABLE 1:** General comparison between group O and group S.

Variable	Group O (n = 80)	Group S (n = 80)	P -value
Sex (male, female)	39/41	37/43	0.752
Age, years	41.5 (34.0, 52.0)	39.0 (32.3, 52.8)	0.751
Height, cm	166.91 ± 8.31	166.52 ± 8.42	0.766
Weight, kg	65.0 (58.0, 72.8)	65.5 (57.3, 73.0)	0.802
BMI, kg/m^2^	23.28 ± 2.48	23.58 ± 2.66	0.459
HR, beats/min	84.4 ± 10.9	82.9 ± 10.2	0.372
ASA classification, I/II	50/30	55/25	0.405
MAP, mmHg	90.4 ± 8.4	91.6 ± 8.9	0.366
SpO_2_, %	100 (99, 100)	100 (99, 100)	0.722
Procedure time, min	13.0 (5.0, 17.8)	12.0 (4.0, 16.0)	0.124
Type of examination, (gastroscopy/colonoscopy/both)	32/7/41	34/5/41	0.821

Data are presented as n (%), mean ± SD, or median (IQR).

Abbreviations: BMI: body mass index; MAP: mean arterial pressure; HR: heart rate; ASA: american society of anesthesiologists; SpO2: pulse oximetry.

#### 3.2.2 Perioperative hemodynamic and respiratory parameters

**FIGURE 5:**
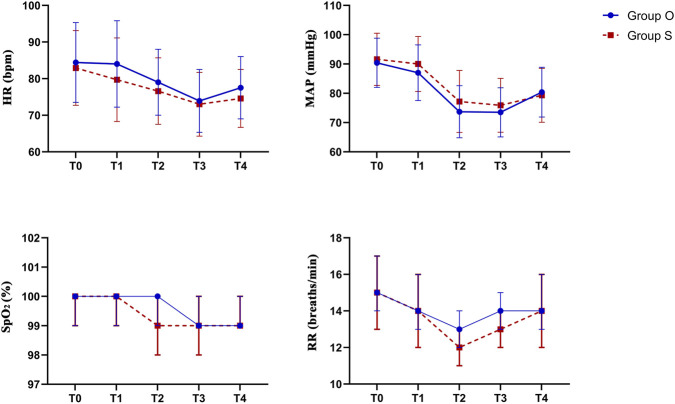
Changes in perioperative hemodynamic and respiratory parameters: Heart rate (HR), Mean arterial pressure (MAP), Pulse oximetry (SpO2), and Respiratory rate (RR). Data are presented as mean ± SD or median (IQR). Blue solid lines with circles represent Group O (oliceridine), and red dashed lines with squares represent Group S (sufentanil). There were no statistically significant differences between the two groups at any time point from T0 to T4 (all *P* > 0.01 after Bonferroni correction for multiple comparisons). T_0_ (baseline, supine position), T_1_ (2 min after opioid administration), T_2_ (endoscope entry into the main anatomical lumen), T_3_ (endoscope withdrawal from the main anatomical lumen), and T_4_ (full recovery of consciousness).

Changes in perioperative monitoring parameters, including MAP, heart rate HR, SpO_2_, and RR at predefined time points (T0 to T4), are illustrated in Table 2; Figure 5. Following the conservative Bonferroni correction for multiple comparisons across five time points (T0–T4), the significance threshold for these specific measurements was strictly set at *P* < 0.01. Under this rigorous criterion, no statistically significant differences were observed between Group O and Group S in MAP, HR, SpO_2_, and RR at any time point (all adjusted *P* > 0.01). While some raw *P*-values for certain parameters (e.g., RR at T2 and T3) were below 0.05, they did not meet the predefined threshold for significance after accounting for multiplicity. Consequently, both oliceridine and sufentanil demonstrated comparable and stable hemodynamic and respiratory profiles throughout the endoscopic procedure.

**TABLE 2:** Changes in perioperative hemodynamic and respiratory parameters.

Measurement indicators/Time points	Group O (n = 80)	Group S (n = 80)	t/Z-value	*P*-value^a^
MAP, mmHg				
T0	90.4 ± 8.4	91.6 ± 8.9	−0.907	0.366
T1	87.0 ± 9.5	90.0 ± 9.4	−1.982	0.049
T2	73.7 ± 8.9	77.2 ± 10.6	−2.264	0.025
T3	73.5 ± 8.4	75.9 ± 9.2	−1.708	0.090
T4	80.4 ± 8.5	79.3 ± 9.2	0.776	0.439
HR, beats/min				
T0	84.4 ± 10.9	82.9 ± 10.2	0.896	0.372
T1	84.0 ± 11.8	79.7 ± 11.4	2.341	0.020
T2	79.0 ± 9.0	76.6 ± 9.1	1.640	0.103
T3	73.9 ± 8.6	73.0 ± 8.7	0.611	0.542
T4	77.5 ± 8.5	74.6 ± 7.9	2.231	0.027
SpO_2_, %				
T0	100 (99, 100)	100 (99, 100)	−0.356	0.722
T1	100 (99, 100)	100 (99, 100)	0.656	0.512
T2	100 (99, 100)	99 (98, 100)	1.876	0.061
T3	99 (99, 100)	99 (98, 100)	2.040	0.041
T4	99 (99, 100)	99 (99, 100)	0.976	0.329
RR, breaths/min				
T0	15 (14, 17)	15 (13, 17)	0.511	0.609
T1	14 (13, 16)	14 (12, 16)	0.715	0.475
T2	13 (12, 14)	12 (11, 13)	2.393	0.017
T3	14 (13, 15)	13 (12, 14)	2.284	0.022
T4	14 (13, 16)	14 (12, 16)	0.629	0.530

Data are presented as mean ± SD, or median (IQR).

^a^ Representing raw *P*-values. According to Bonferroni correction for multiple comparisons (5 time points), the adjusted alpha threshold for statistical significance is set at *P* < 0.01. Therefore, all *P-*values in the above table were >0.01, indicating no statistically significant differences between the two groups.

Abbreviations: MAP: mean arterial pressure; HR: heart rate; SpO_2_: pulse oximetry; RR: respiratory rate.

#### 3.2.3 Postoperative recovery profiles

Patients in Group O exhibited a significantly faster and higher-quality postoperative recovery compared to those in Group S (Table 3). The median time to the first attainment of a modified Aldrete score ≥9 was significantly shorter in Group O (5.0 [4.0, 7.0] min) than in Group S (6.0 [4.0, 9.0] min, *P* = 0.013). Consequently, the median discharge time was also significantly reduced in Group O (13.0 [11.0, 16.0] min vs. 15.0 [12.0, 17.0] min, *P* = 0.044). Additionally, postoperative pain assessment revealed that patients in Group O experienced significantly lower VAS scores (1.0 [1.0, 1.0]) compared to Group S (1.0 [1.0, 2.0], *P* = 0.009), indicating an optimized analgesic profile.

**TABLE 3:** Comparison of the indicators between group O and group S.

Variable	Group O (n = 80)	Group S (n = 80)	χ2/Z/t- value	*P* -value
Total propofol dose, mg	197.5 ± 52.6	192.9 ± 52.1	0.553	0.581
Injection pain	10 (12.5)	10 (12.5)	0.000	1.000
Respiratory depression	6 (7.5)	16 (20.0)	4.269	0.039*
Hemodynamic adverse events	13 (16.25)	11 (13.75)	0.049	0.825
Hypotension	8 (10.0)	6 (7.5)	0.078	0.780
Bradycardia	5 (6.25)	5 (6.25)	0.000	1.000
PONV	2 (2.5)	5 (6.3)	—	0.443
Dizziness	2 (2.5)	3 (3.8)	—	1.000
VAS score	1.0 (1.0, 1.0)	1.0 (1.0, 2.0)	−2.606	0.009*
Time to first attainment of a modified aldrete score ≥9, min	5.0 (4.0, 7.0)	6.0 (4.0, 9.0)	−2.495	0.013*
Discharge time, min	13.0 (11.0, 16.0)	15.0 (12.0, 17.0)	−2.019	0.044*

Data are presented as mean ± standard deviation (SD), median (interquartile range) [IQR], or n (%). Categorical variables, including the incidence of respiratory depression, injection pain, and hemodynamic advers events (hypotension and bradycardia), were analyzed using Pearson’s chi-square test with Yates’ continuity correction, as all expected cell counts in these comparisons were ≥5. Fisher’s exact test was applied for PONV, and dizziness due to small expected cell counts (<5). Intergroup comparisons for non-normally distributed continuous variables (VAS, score, recovery times) were performed using the Mann-Whitney U test. *Indicates *P* < 0.05 compared with Group S.

Abbreviations: VAS: visual analogue scale; PONV:postoperative nausea and vomiting.

#### 3.2.4 Adverse events

The incidences of perioperative adverse events are summarized in Table 3. The most notable finding was a significantly lower incidence of respiratory depression in Group O (7.5%, 6/80) compared to Group S (20.0%, 16/80) (*P* = 0.039). The occurrences of other adverse events, including injection pain (12.5% vs. 12.5%, *P* = 1.000), hemodynamic adverse events (16.25% vs. 13.75%, *P* = 0.825), hypotension (10.0% vs. 7.5% *P* = 0.780), bradycardia (6.25% vs. 6.25% *P* = 1.000), PONV (2.5% vs. 6.3%, *P* = 0.443), and dizziness (2.5% vs. 3.8%, *P* = 1.000), were comparable between Group O and Group S, with no statistically significant differences observed.

## 4 Discussion

In this two-phase investigation, we derived the ED_95_ for oliceridine and sufentanil in endoscopic analgesia and evaluated clinical outcomes at these predefined doses under tightly controlled propofol sedation. Within this standardized framework, oliceridine was associated with a lower observed incidence of respiratory depression and modestly faster recovery compared with sufentanil. The absolute difference in discharge time was small, suggesting that any clinical advantage is more likely related to safety rather than efficiency.

### 4.1 Primary efficacy (recovery and respiratory safety)

Prolonged patient stay in the PACU is a critical issue that creates bottlenecks, potentially delaying subsequent surgical schedules and increasing institutional costs (Lalani et al., 2013; Akabane et al., 2025). Based on motor response suppression as a comparable clinical endpoint, this randomized trial utilized oliceridine and sufentanil at their respective ED_95_ doses (14.52 μg/kg vs. 0.048 μg/kg). Our results establish that oliceridine provides a superior therapeutic index regarding recovery efficiency and respiratory safety. However, while oliceridine statistically shortened the time to discharge compared to sufentanil (13.0 min vs. 15.0 min; *P* = 0.044), this absolute difference of 2 min is relatively modest. Consequently, its direct impact on alleviating the aforementioned bottlenecks or optimizing resource utilization in routine clinical practice may be limited. Instead, the core clinical value of oliceridine demonstrated in this study lies in its superior respiratory safety profile. Under standardized dose based on ED_95_ derived from phase 1, the incidence of respiratory depression was significantly lower (7.5% vs. 20.0%) in the oliceridine group (7.5% vs. 20.0%; *P* = 0.039). Although a conservative sufentanil dose (0.048 μg/kg) was used, the 20% respiratory event rate underscores a critical pharmacological challenge. According to the interaction models established by Vuyk (1997), propofol not only acts synergistically with opioids but also inhibits the metabolism of sufentanil, thereby elevating its plasma concentration. This interaction likely intensifies the synergistic suppression of the hypercapnic ventilatory drive, explaining the high incidence of respiratory depression even at sub-maximal dosages. The incidence of respiratory depression in our oliceridine group was remarkably low (7.5%). While a recent large-scale randomized controlled trial by Ma et al. (2025), also confirmed the safety advantages of oliceridine over sufentanil during GI endoscopy, they reported a considerably higher overall incidence (14.1% vs. 21.8%). We attribute the remarkably lower event rate in our study to the following reasons.

### 4.2 Mechanistic basis

The enhanced safety demonstrated in this study is consistent with oliceridine’s unique pharmacokinetic and pharmacodynamic profile. From a pharmacokinetic perspective, oliceridine is reported to minimize the redistribution and tissue accumulation typical of highly lipophilic opioids like sufentanil. It is metabolized predominantly via CYP3A4 and CYP2D6 into inactive compounds, potentially avoiding the prolonged sedative effects and delayed recovery often associated with conventional potent opioids (Mercadante, 2019; Daksla et al., 2023). Pharmacodynamically, this mechanism is hypothesized to be multi-layered. Oliceridine is characterized as a G protein-biased ligand favoring analgesic signaling over β-arrestin2 recruitment (Simons et al., 2023; Soergel et al., 2014b; Singla et al., 2017), while also displaying low intrinsic efficacy at the μ-opioid receptor (He et al., 2021; Hill et al., 2018; Gillis et al., 2020). This partial agonist profile may create a functional ceiling on respiratory depression, suggesting it provides adequate analgesia to blunt procedural stimuli without engaging the extensive receptor reserve needed to induce severe respiratory depression or apnea. While our study did not directly measure these molecular pathways, the clinical findings appear to align with the synergy between oliceridine’s biased signaling pathway and its limited receptor activation.

### 4.3 Dosing consistency and sedative synergy

Our determined ED_95_ of oliceridine (14.52 μg/kg) combined with propofol is highly consistent with the findings of Cao et al. (2025), who reported an ED_95_ of 14.46 μg/kg for males and 13.19 μg/kg for females in a similar gastroscopy setting. This concordance reinforces the reliability of our dose-finding methodology. Interestingly, our required ED_95_ is notably lower than the 18.82 μg/kg reported by Yang et al. (2025). This discrepancy is likely explained by their co-administration of oliceridine with remimazolam (0.3 mg/kg) rather than propofol. This suggests that the synergistic pharmacodynamic interaction and opioid-sparing effect of propofol may be stronger than that of remimazolam, necessitating tailored oliceridine dosing strategies based on the specific co-administered sedative agent.

### 4.4 Objective standardization of sedation depth

A fundamental methodological strength of the current study, which distinguishes it from recent literature, is the implementation of Bispectral Index (BIS) monitoring. Previous trials investigating oliceridine in endoscopic settings including those by Cao, Yang, and Ma have predominantly relied on subjective clinical scales, such as the OAA/S or MOAA/S (Ma et al., 2025; Cao et al., 2025; Yang et al., 2025). Such observational assessments are inherently intermittent and susceptible to inter-observer variability, which can inadvertently lead to excessive sedation. Furthermore, we utilized a precisely calculated ED_95_ dose, mitigating the potential over-titration associated with the fixed empirical doses (e.g., 1 or 1.5 mg) used by Ma et al. (2025). In our investigation, BIS values were strictly maintained between 50 and 60. By meticulously eliminating the confounding influence of fluctuating sedation depths, we successfully averted deep sedation-induced hypoventilation. Consequently, we can confidently attribute the observed differences in respiratory safety directly to the distinct receptor-binding properties of the respective opioids.

### 4.5 Secondary safety and hemodynamics

The overall incidence of hemodynamic adverse events, defined as either hypotension or bradycardia, was 16.25% (13/80) in Group O and 13.75% (11/80) in Group S. No statistically significant difference was observed between the two groups (*P =* 0.825). Specifically, the incidence of hypotension was 10.0% vs. 7.5% (*P =* 0.780), and bradycardia was 6.25% in both groups (*P =* 1.000). This clinical trend aligns with the previously reported favorable hemodynamic profile of oliceridine, suggesting minimal impact on cardiovascular compensatory mechanisms (Dahan et al., 2020; Singla et al., 2019). While VAS scores differed statistically between groups, both provided adequate analgesia with a small absolute difference, suggesting limited clinical relevance.

Adverse events such as PONV and dizziness were infrequent and did not differ significantly between groups. The lower-than-expected PONV incidence contrasts with some prior reports (Daksla et al., 2023). The brief procedure duration and low cumulative opioid dose in this diagnostic setting may explain this discrepancy.

From a cost perspective, although oliceridine carries a higher initial drug cost, modeling studies indicate that this may be offset by reduced expenses from fewer respiratory adverse events and earlier discharge (Simpson et al., 2021; Simpson et al., 2022). The lower incidence of respiratory depression with oliceridine reduces the need for emergency airway interventions (such as jaw thrust or mask ventilation) during clinical management, optimizing perioperative workflows and making it a preferable choice in busy outpatient settings.

### 4.6 Limitations

We acknowledge several limitations in this study. First, while the Dixon up-and-down method is standard for estimating the ED_50_, its inherent statistical properties limit precision when calculating extreme percentiles like the ED_95_, resulting in a relatively wide confidence interval for oliceridine. Future studies could therefore consider adopting a biased-coin design to improve allocation efficiency and potentially achieve greater precision (Oron et al., 2022).

Second, we acknowledge an inherent limitation in our power analysis. Although the study was primarily powered to detect differences in clinical efficiency (discharge time), the observed difference in respiratory depression (7.5% vs. 20.0%) still yielded a post-hoc power of approximately 63%. While this power is below the conventional 80% threshold, the statistical significance achieved (*P* = 0.039) underscores a clinically meaningful safety signal. This suggests that oliceridine’s respiratory safety benefits are pronounced enough to be detected even within a sample size optimized for efficiency metrics.

Third, excluding therapeutic procedures and ASA III–IV patients limits the generalizability of the findings. However, these high-risk populations, who are most susceptible to opioid-induced respiratory depression, might benefit the most from oliceridine’s respiratory ceiling effect.

Fourth, a significant methodological limitation is the absence of capnometry [end-tidal carbon dioxide (EtCO_2_) monitoring] for the continuous assessment of ventilatory status. While respiratory rate was monitored via thoracic impedance and clinical observation, capnometry is considered the gold standard for the early detection of respiratory depression during procedural sedation. The lack of, EtCO_2_ monitoring might have led to an underestimation of transient or obstructive respiratory events that do not immediately result in oxygen desaturation. We attempted to mitigate this risk by maintaining a strictly standardized depth of sedation using Bispectral Index (BIS) monitoring; however, future research should incorporate capnography to ensure more sensitive and comprehensive safety monitoring.

Fifth, although we found statistical significance in recovery metrics, the absolute clinical differences—such as the 2-min reduction in discharge time—are relatively modest. Therefore, these efficiency gains should be interpreted with caution in terms of their impact on overall institutional workflow. The more clinically meaningful advantage of oliceridine demonstrated here is its significantly lower incidence of respiratory depression at equipotent doses.

Sixth, our anesthesia protocol utilized continuous propofol infusion maintained at a standardized BIS level (50–60) rather than the intermittent boluses common in procedural sedation. While this objective standardization was vital for isolating the pharmacological effects of the study drugs, continuous infusion may have narrowed the gap in total propofol consumption between the two groups. This standardized approach might have masked potential opioid-sparing effects of oliceridine that could be more evident under intermittent dosing strategies. Future studies could explore whether different propofol administration methods influence the synergistic profile and total sedative requirements. Finally, the monitoring window concluded at discharge, which could have overlooked delayed adverse events, particularly PONV. Thus, although these results position oliceridine as a compelling alternative for healthy adults, additional validation in diverse, high-risk cohorts with adequately powered sample sizes is needed to fully delineate its role in endoscopic practice.

## 5 Conclusion

At estimated ED_95_ doses derived from Phase one, oliceridine combined with propofol provides effective conditions for GI endoscopy with a significantly lower risk of respiratory depression and faster recovery compared to sufentanil. Oliceridine represents a promising alternative for analgesia in ambulatory settings where safety and efficiency are paramount.

## Data Availability

The data that support the findings of this study are available from the corresponding author upon reasonable request.
